# *Yarrowia lipolytica*: the novel and promising 2-phenylethanol producer

**DOI:** 10.1007/s10295-013-1240-3

**Published:** 2013-02-27

**Authors:** E. Celińska, P. Kubiak, W. Białas, M. Dziadas, W. Grajek

**Affiliations:** 1 Department of Biotechnology and Food Microbiology, Poznan University of Life Sciences, ul. Wojska Polskiego 48, 60-627 Poznan, Poland; 2 Institute of Food Technology, Poznan University of Life Sciences, ul. Wojska Polskiego 31, 60-624 Poznan, Poland

**Keywords:** *Yarrowia lipolytica*, 2-phenylethanol, Aroma compound, Bioconversion

## Abstract

This is the first report on the ability of *Yarrowia lipolytica* strains to produce 2-phenylethanol (2-PE), which has not been identified for this species to date. 2-PE is a valuable aroma compound of rose-like odor. Its isolation from the other than microbial source—rose petals, is limited by the substrate availability. Thus, this chemical compound constitutes an attractive product for biotechnological conversions. To date, the ability to produce 2-PE has been described for such genera as *Saccharomyces* sp., *Kluyveromyces* sp., *Geotrichum* sp., and *Pichia* sp. This report provides evidence that *Y. lipolytica* is a novel 2-PE producer. Moreover, the titers of 2-PE obtained in *Y. lipolytica* NCYC3825 non-optimized cultures, nearly 2 g/l, are competitive to titers obtained by the other species.

## Introduction

*Yarrowia lipolytica* is a dimorphic, nonconventional yeast species with unique metabolic properties. Its GRAS status and exceptional performance in utilization of different raw biomaterials and their bioconversion into high-value added bioproducts stimulates its frequent exploitation in industrial applications.

2-phenylethanol (2-PE) is an aroma compound that is widely used in cosmetics and food production. 2-PE of natural origin is particularly desired by the consumer, which is reflected in a dramatic divergence in price: 1 kg of 2-PE produced by traditional chemical synthesis from benzene and styrene costs USD 3.5, and 2-PE produced by natural routes, from rose petals or from microbiological bioconversion, costs USD 1,000/kg [8]. In the literature, several microbial species have been described as possessing the ability to synthesize 2-PE (as reviewed in [5]). In yeast cells, 2-PE can be synthesized via two independent routes, either de novo or as a result of bioconversion of l-phenylalanine (l-Phe). In the yeast cells, the l-Phe bioconversion route (Ehrlich pathway [4]) represents the more efficient and thus more industrially attractive option.

This report provides evidence of a novel industrially attractive metabolic trait of *Y. lipolytica*, namely, production of relatively high titers of 2-phenylethanol.

## Materials and methods

### Strains

The *Yarrowia lipolytica* NCYC3825 strain used in this study was deposited in the National Collection of Yeast Cultures (Norwich, UK). The strain was genetically engineered as described in [2]. *Y. lipolytica* strains B57-4, A18 and culture supernatants for HPLC analysis of A101.1.31, A15, MK1, and A101 strains were kindly donated by Prof. W. Rymowicz and Prof. M. Robak from Wroclaw University of Environmental and Life Sciences.

### Media and culture conditions

Routine laboratory cultivation of *Y. lipolytica* strains was carried out in YPD and MMT media, in shake flasks. Comparison of efficiency of the 2-PE production was carried out in shake flasks in the medium containing 10 g of yeast extract and 40 g of glucose per liter of tap water (30 °C, 250 rpm). Bioconversion of l-Phe to 2-PE was carried out in shake flasks in the cultivation medium (modified, based on [3]) containing per liter of deionized water: glucose 40 g, KH_2_PO_4_ 15 g, MgSO_4_·7H_2_O 0.5 g, YNB 0.2 g, trace elements solution according to [1] 1 ml, thiamine 3 mg, pH 6.5, supplementation with l-Phe 7 g/l after 73 h of cultivation. Bioconversion of l-Phe to 2-PE in the bioreactor cultures was carried out in the presence of glycerol (50 ml/l) as a carbon source—remaining medium components as in the shake flask culture experiments, with l-Phe 8 g/l contained in the bioconversion medium (pH 4.0, at 30 °C, 3 vvm of air, aeration and stirring adjusted automatically to maintain 30 % saturation of oxygen dissolved in the culture). Bioreactor was inoculated with 73 h culture of *Y. lipolytica* NCYC3825. Biomass buildup was monitored by the optical density measurement at 600-nm wavelength.

### Analytical methods

#### GC/MS: identification of 2-PE

Culture supernatants filtered through 0.45 μm syringe filter were transferred onto an activated SPE-C18 cartridge (500 mg/3 ml, Thermo Scientific). Extraction was carried out with vacuum SPE station at a flow rate of 2 ml/min. Following extraction, the column was washed with 20 ml of deionized water. The non-polar fraction was eluted with 5 ml of pentane: dichloromethane mix (2:1 (v/v)), and dried with Na_2_SO_4_. The extract was concentrated to 500 μl and analyzed using GC/MS. GC/MS analysis was carried out with an Agilent 7890A gas chromatograph coupled to a single-quadrupole 5975C VL MSD equipped with a Supelcowax capillary GC column (60 m × 0.2 mm × 0.2 μm; Sigma Aldrich, St. Louis, MO, USA). Mobile phase: helium at a flow rate of 0.8 ml/min. Inlet port temperature 290 °C. Oven program: 1 min: 40 °C followed by 8 °C/min to 180 °C for 1 min, 1 min: 200 °C, 20 min: 240 °C. Mass detector parameters: transfer line 230 °C, scan mode in a range of 33–488 *m/z*, electron impact ionization at 70 eV.

#### HPLC: determination of 2-PE and l-Phe concentration

Culture supernatants filtered through 0.45 μm syringe filter were analyzed with an Agilent 1200 liquid chromatograph equipped with a DAD detector (254 nm) and a LiChroCART 125-4 Superspher 100 RP-18e (4 μm; MerckMillipore) column. Gradient elution at a flow rate of 1 ml/min was performed as follows: 0 min-5 % of component B, 10 min-65 %B, 11 min-100 %B, 15 min-100 %B, 18 min-5 %B, 23 min- %B. Mobile phase components: A—0.01 M HCl, B—80:20 (acetonitrile: 0.025 M HCl). The column temperature was 40 °C. Quantitative and qualitative identification of 2-PE and l-Phe was carried out with an external standard method, using peak surface for calculations.

## Results and discussion

**Fig. 1:**
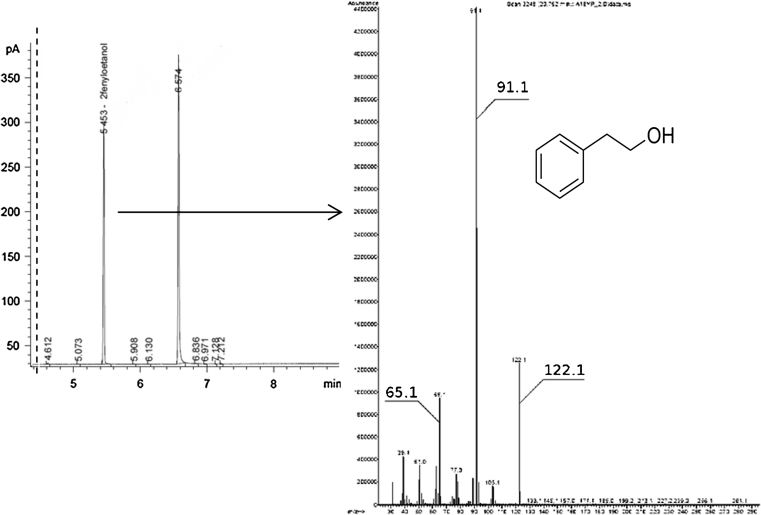
GC/MS analysis indicating presence of 2-PE in *Y. lipolytica* NCYC3825 culture supernatants. Mass spectrum of 2-PE, which allowed identification of the compound

GC/MS analysis of *Y. lipolytica* NCYC3825 culture supernatants from our preliminary experiments, where l-Phe was not externally supplemented into the medium, surprisingly showed that the strain produces 2-PE (chromatogram and mass spectrum of 2-PE are shown in Fig. 1). Since the ability to produce 2-PE has not been earlier described for *Y. lipolytica*, further experiments were set up in order to elucidate the potential of *Y. lipolytica* to produce this metabolite.

First, six different *Y. lipolytica* strains (A101.1.31, A18, A15, B57-4, MK1, A101) were cultured in order to verify if the ability to produce 2-PE is strain-dependent. In four culture supernatants of the analyzed strains, 2-PE was detected: A101.1.31: 0.24 g/l, A18: 0.02 g/l, B57-4: 0.04 g/l, A15: 0.01 g/l. For two other natural isolates: MK1 and A101—2-PE was not detected. Thus, it appeared that the ability to produce 2-PE at the detectable level is not uniform among these *Y. lipolytica* strains and the efficiency of the production is strain-dependent. This statement corresponds well with the literature data, where different yeast strains and species were screened for 2-PE production [7].

**Fig. 2:**
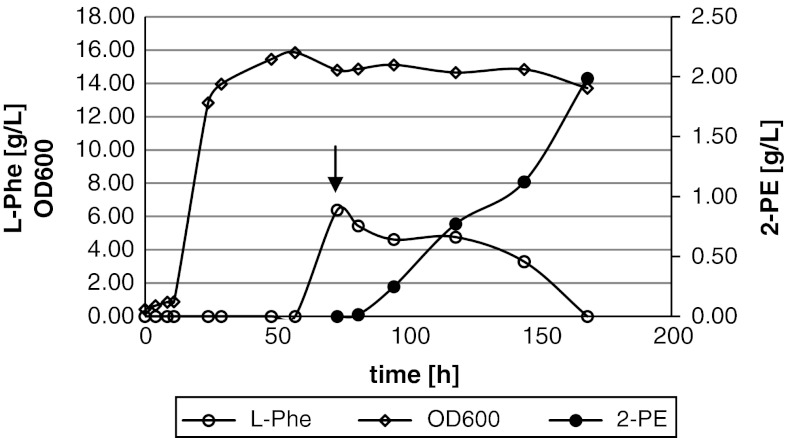
l-Phe utilization, 2-PE, and biomass production during *Y. lipolytica* NCYC3825 cultivation in shake flasks in the presence of glucose as a carbon source. The *arrow* indicates the time of l-Phe addition. *Open diamonds*—biomass formation OD600, *open circles*—l-Phe concentration, *filled circles*—2-PE concentration. l-Phe was supplemented into the culture after 73 h, when the culture reached late stationary phase

Subsequently, 2-PE formation kinetics in the presence of glucose was determined for the best identified *Y. lipolytica* 2-PE producer, NCYC3825. Since in other yeast species 2-PE is primarily formed as a product of l-Phe bioconversion via the Ehrlich pathway, l-Phe was contained in the media used for cultivation tests. l-Phe utilization, 2-PE, and biomass production kinetics curves are presented in (Fig. 2). After 95-h cultivation starting from l-Phe addition, all l-Phe was utilized and the final titer of 2-PE reached 1.98 g/l, with productivity and bioconversion rate reaching 20 mg/(L × h) and 0.31 g/g, respectively.

**Fig. 3:**
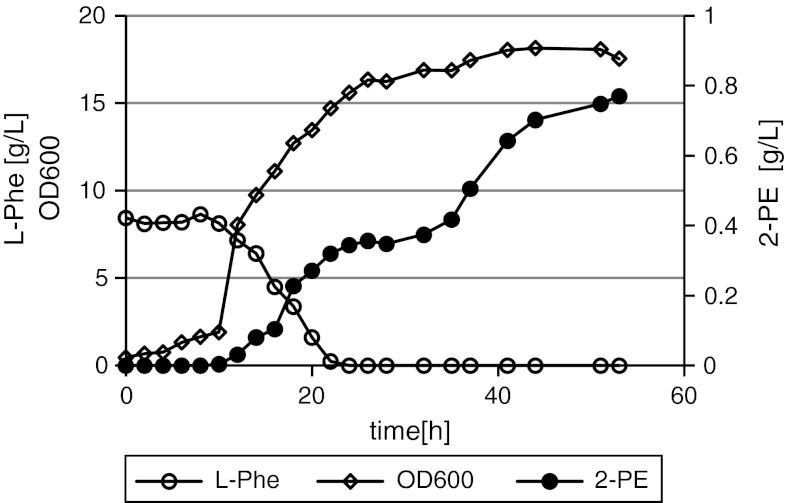
l-Phe utilization, 2-PE, and biomass production during *Y. lipolytica* NCYC3825 cultivation in a bioreactor in the presence of glycerol as a carbon source (50 ml/l). *Open diamonds*—biomass formation OD600, *open circles*—l-Phe concentration, *filled circles*—2-PE concentration. l-Phe was contained in the cultivation medium from the time 0

In order to investigate if *Y. lipolytica* NCYC3825 exhibits the ability to produce 2-PE in the presence of other carbon source, the bioreactor culture with glycerol as the carbon source was set up. l-Phe utilization, 2-PE, and biomass production kinetics curves are presented in (Fig. 3). All l-Phe was utilized after 24 h of cultivation, and the final titer of 2-PE reached 770 mg/l after 54 h, with the productivity and the bioconversion yield reaching 14.52 mg/(L × h) and 0.09 g/g l-Phe, respectively. The low bioconversion yield of l-Phe to 2-PE in the bioreactor culture in the presence of glycerol can be probably attributed to utilization of l-Phe in the cinnamate pathway, which constitutes one of several possibilities of l-Phe degradation in yeast cells. l-Phe was probably used by fast-growing cells, which were in the logarithmic phase of growth between 10 and 24 h after inoculation (Fig. 3). Furthermore, it seems that in *Y. lipolytica* cells, the activity of the first enzyme of the cinnamate pathway, phenylalanine ammonia lyase, is inhibited in the presence of glucose, as it was shown for *Rhodotorula glutinis* [10]. This statement is corroborated with the observation that similar pattern of l-Phe supplementation (addition at the beginning of culturing) into the medium containing glucose as a carbon source (data not shown), brought relatively high bioconversion yield 0.26 g/g, compared to 0.09 g/g when glycerol was used as a carbon source. Though, it should be noted that the activities involved in the cinnamate pathway were not identified and described for *Y. lipolytica* to date, thus, these considerations are speculative. To eliminate l-Phe dissipation in “non-productive” pathways when cultured on glycerol, a different strategy of l-Phe supplementation should be applied.

The maximum bioconversion rate that was obtained with *Y. lipolytica* (0.31 g/g) still significantly differs from the maximum theoretical bioconversion yield of 0.75 g/g [5]. Comparison of the obtained final titers of 2-PE with the literature data indicates that *Y. lipolytica* shows the potential to efficiently produce this metabolite. For example, *A. niger* produced 1.4 g/l 2-PE from 6 g/l l-Phe in 9 days [11], *P. fermentans* L-5 produced 0.5 g/l 2-PE from 1 g/l l-Phe in 16 h [9], strains of *K. marxianus* produced up to 0.9 g/l of 2-PE [7] in a non-optimized process, *K. marxianus* CBS600 produced 5.6 g/l of 2-PE after medium optimization [6]. In the scope of the above, the process of 2-PE production with *Y. lipolytica* appears promising, however it requires further research and optimization.

## References

[1] Barth G, Gaillardin C, Wolf K (1996). *Yarrowia lipolytica*. Nonconventional yeasts in biotechnology. A Handbook.

[2] Celińska E (2012) Construction and characterization of *Yarrowia lipolytica* recombinant strain bearing heterologous genes of glycerol catabolism. PhD Thesis, Poznan University of Life Sciences

[3] Cui Z, Yang X, Shen Q, Wang K, Zhu T (2011). Optimisation of biotransformation conditions for production of 2-phenylethanol by a *Saccharomyces cerevisiae* CWY132 mutant. Nat Prod Res.

[4] Ehrlich F (1907). Über die Bedingungen der Fuselölbildung und über ihren Zusammenhang mit dem Eiweißaufbau der Hefe. Ber Dtsch Chem Ges.

[5] Etschmann MM, Bluemke W, Sell D, Schrader J (2002). Biotechnological production of 2-phenylethanol. Appl Microbiol Biotechnol.

[6] Etschmann MM, Sell D, Schrader J (2004). Medium optimization for the production of the aroma compound 2-phenylethanol using a genetic algorithm. J Mol Catal B Enzymatic.

[7] Etschmann MM, Sell D, Schrader J (2003). Screening of yeasts for the production of the aroma compound 2-phenylethanol in a molasses-based medium. Biotechnol Lett.

[8] Hua D, Xu P (2011). Recent advances in biotechnological production of 2-phenylethanol. Biotechnol Adv.

[9] Huang CJR, Lee SL, Chou CC (2000). Production and molar yield of 2-phenylethanol by *Pichia fermentans* L-5 as affected by some medium components. J Biosci Bioeng.

[10] Large PJ (1986). Degradation of organic nitrogen compounds by yeasts. Yeast.

[11] Lomascolo A, Lesage-Meessen L, Haon M, Navarro D, Antona C, Faulds C, Marcel A (2001). Evaluation of the potential of *Aspergillus niger* species for the bioconversion of l-phenylalanine into 2-phenylethanol. World J Microbiol Biotechnol.

